# Environmental tobacco smoking (ETS) and esophageal cancer: A population‐based case‐control study in Jiangsu Province, China

**DOI:** 10.1002/ijc.35254

**Published:** 2024-11-18

**Authors:** Zi‐Yi Jin, Kuangyu Liu, Gina Wallar, Jin‐Yi Zhou, Li‐Na Mu, Xing Liu, Li‐Ming Li, Na He, Ming Wu, Jin‐Kou Zhao, Zuo‐Feng Zhang

**Affiliations:** ^1^ Department of Epidemiology, School of Public Health Fudan University Shanghai China; ^2^ Nanjing Drum Tower Hospital Clinical College of Nanjing Medical University Nanjing Jiangsu China; ^3^ Department of Epidemiology, Fielding School of Public Health University of California Los Angeles California USA; ^4^ Department of non‐communicable diseases prevention and control Jiangsu Provincial Center for Disease Control and Prevention Nanjing Jiangsu China; ^5^ Department of Social and Preventive Medicine, School of Public Health and Health Professions University at Buffalo, The State University of New York Buffalo New York USA; ^6^ Department of Epidemiology, School of Public Health Peking University Beijing China

**Keywords:** case–control study, China, environmental tobacco smoking, esophageal cancer

## Abstract

Esophageal cancer continues to pose a significant public health issue in areas with increased incidence rates such as China. Although involuntary smoking was defined as a group 1 carcinogen for lung cancer, few studies have explored the impact of environmental tobacco smoking (ETS) on esophageal cancer. In this paper, we examined the association between ETS and esophageal cancer in high‐risk groups in Jiangsu Province, China. Epidemiologic data were collected for 2969 newly diagnosed cases and 8019 population controls including exposure to active/passive smoking and risk factors. The unconditional logistic regression model and the semi‐Bayes (SB) method were applied to assess adjusted odds ratios (ORs) and confidence intervals (CIs). ETS exposure (ever vs. never) was positively associated with esophageal cancer with an SB‐adjusted OR (95% CI) of 1.44 (1.31–1.58) among overall population, and 1.56 (1.35–1.82) among non‐smokers (i.e., non‐active smokers), with corresponding population attributable fractions of 15.0% (95% CI: 10.3%–18.9%) and 12.1% (95% CI: 8.8%–19.8%), respectively. The association was more prominent in men at work and in women at home, with SB‐adjusted OR (95% CI) of 1.36 (1.17–1.58) and 1.61 (1.35–1.58), respectively. A dose–response relationship between ETS exposure and the disease was detected across the entire population as well as in non‐smokers. This is the largest population‐based case–control study of ETS and esophageal cancer and the first study to evaluate such association among non‐smokers in a Chinese population. We recommend strengthening the ongoing anti‐tobacco public health initiatives in China with a particular emphasis on creating a tobacco‐free work/home environment.

## Graphical abstract

What's new?

Although passive smoking has been defined as a group 1 carcinogen for lung cancer, few studies have explored the impact of environmental tobacco smoking on esophageal cancer. In this large Chinese population‐based case–control study, environmental tobacco smoking consistently showed a positive relationship with esophageal cancer in the overall population and among non‐smokers, affecting both men and women. About 15.0% of cases in the overall population were attributable to environmental tobacco exposure both at home and in the workplace. A more comprehensive smoke‐free law would be an effective prevention strategy to reduce esophageal cancer incidence in China.

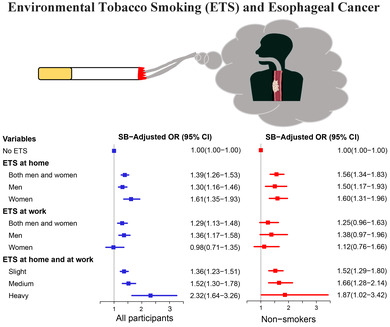

## 1 INTRODUCTION

Esophageal cancer ranks as the eighth most prevalent and the sixth deadliest cancer globally.^1^, ^2^ Eighty percent of esophageal cancer cases globally originate in developing countries, where it is considered the fourth most frequently diagnosed malignancy and the second leading cause of death due to oncogenic diseases.^3^ Eastern Asia as well as Eastern and Southern Africa suffer from the highest mortality rates.^4^ Asia exhibits incidence rates up to 20 times higher compared to regions like the United States, where the risk remains significantly lower. Squamous cell esophageal cancer, the pathologic type predominantly found in areas with high incidence rates including Asia, has been primarily associated with a combination of tobacco and alcohol use.^5^, ^6^, ^7^, ^8^, ^9^

Studies have established a strong link between tobacco smoking and an elevated risk for esophageal cancer.^10^, ^11^, ^12^ Specifically, the risk associated with smoking can increase from twofold to tenfold depending on the study design and sample size. The risk of esophageal cancer is modulated by both the intensity and the duration of smoking; however, empirical evidence suggests that the duration of exposure exerts a more significant influence than the intensity.^13^ Furthermore, alcohol consumption has been identified to have a multiplicative interaction with smoking.^5^ Castellsague et al., in a meta‐analysis on smoking and esophageal cancer, discovered that the risk of esophageal cancer correlates with the duration and quantity of both smoking and alcohol consumption in a dose–response manner.^6^

Environmental tobacco smoke (ETS) is posited to be linked with an elevated risk of tobacco‐associated malignancies. Extensive research on ETS and lung cancer has unveiled a consistently higher risk.^14^, ^15^ As a result, it has been classified as a group I carcinogen specifically for lung cancer by the International Agency for Research on Cancer (IARC).^16^ Nonetheless, the potential relationship between ETS exposure and esophageal cancer within the Chinese population remains underexplored, with only limited findings from two small case–control studies indicating such an association.^17^, ^18^ In this large Chinese population‐based case–control study, we examined the association of ETS exposure with esophageal cancer and explored the link across the general population, as well as among only non‐smokers. We estimated adjusted odds ratios (OR) and related population attributable fraction (PAF) for ETS to understand the impact of ETS on esophageal cancer risk in the Chinese population.

## 2 MATERIALS AND METHODS

### 2.1 Study design and participants

Methods for this research have been detailed previously.^19^ Briefly, data were collected in Dafeng, Ganyu, Chuzhou, and Tongshan which are rural areas situated in the northern part of Jiangsu province, in southeastern China. We included newly identified primary esophageal cancer cases from the counties' cancer registries from 2003 to 2010. Most cases were diagnosed through pathology (41.8%) and endoscopy (30.2%). Other diagnostic methods included medical imaging (21.5%), clinical diagnosis (5.3%), and biochemistry (1.1%).^19^ Only new cases diagnosed within 1 year were recruited in this study and were classified according to the International Classification of Diseases, 10th Revision (ICD‐10, codes C15.0 to C15.9). The morphological verification rate of esophageal cancer from the overall Jiangsu cancer registry was 58.4%, which was higher than 41.8% in this study. It was mandatory for all regional hospitals to report newly identified esophageal cancer cases to the local Center for Disease Control and Prevention. We observed a recruitment rate of 30% among patients registered with esophageal cancer in four counties. Population‐based controls were selected from the healthy individuals from each county with a 1:1 ratio to cases of four types (esophageal, stomach, lung, and liver cancers). They were randomly selected from the residence list based on the county demographic database and needed to be in good health condition to ensure the reliability of their responses.^7^ Controls were individually matched on age (±5 years old), sex, and residential place. We have used a combined control group with all controls for these four cancer types in this analysis to retain statistical power. During the phase of participant recruitment, the rate of response among healthy population controls for this study approximated 87%. Participants were eligible if they had resided in the study area for a minimum of 5 years and had no prior cancer diagnoses. The study included 2969 cases and 8019 controls.

### 2.2 Epidemiological data collection

Epidemiologic data on demographic characteristics, and lifestyle habits including both direct and indirect exposure to tobacco smoke and alcohol consumption, familial cancer history, dietary patterns, and occupational and additional environmental exposures were collected through face‐to‐face interviews using one standardized questionnaire. Details regarding the pathological diagnosis were acquired through consultations with pathologists or from medical documentation. ETS data were collected by the following questions: (1) Did anyone in your household (including parent, spouse, other relatives, and boarders) smoke in your presence? (2) In your place of work, were you exposed to other people's tobacco smoke for more than 15 min a day in closed areas? They were then asked about the degree of exposure of the ETS as light (1–2 days/week), moderate (3–5 days/week), or heavy (every day). Participants were also interviewed regarding the duration of their lifetime exposure (in years) to potential passive smoking. By multiplying the exposure duration by the exposure degree, we calculated cumulative exposure intensity. This was then divided into five groups: no exposure to ETS and four additional groups based on the quartiles of exposure intensity to ETS in the control group.

### 2.3 Statistical analysis

Exposure was defined as any reported ETS at home or workplace or both while non‐exposure was defined as no ETS at either home or workplace. For the purpose of analyzing the dose–response relationship, exposure levels were assigned as follows: 0 for no exposure, 1 for light exposure, 2 for moderate exposure, and 3 for heavy exposure at each source (work and home) in the separate analyses of exposure at either location. When both exposures at home and work were combined, the scores were summed to a total possible maximum of 6. For the combined analysis, scores of 1–2 were considered as light, 3–4 as moderate, and 5–6 as heavy exposure. The dose–response relationship was assessed by treating the degrees of ETS exposure (0, 1, 2, 3) as a continuous variable in a logistic regression model. We adjusted for the following factors: gender (male = 1 and female = 0; except when stratified by gender), age (continuous), education (illiteracy = 1, primary = 2, middle = 3, high or college = 4), income 10 years ago (Yuan/year, continuous), body mass index (BMI, continuous), family history of esophageal cancer (yes = 1, no = 0), county of residence (Dafeng = 1, Ganyu = 2, Chuzhou = 3, Tongshan = 4), pack‐year of smoking (continuous), and ethanol consumption (mL/week, continuous). We calculated the crude and adjusted ORs, together with their 95% confidence intervals (CI) by fitting unconditional logistic regression models. In order to reduce false‐positive findings, we generated posterior estimates that combine observed data associations with null prior associations (Prior OR = 1.00, 95% CI: 0.25–4.00) using the semi‐Bayes (SB) method.^20^ Adjusted population‐attributable fractions (PAFs) were determined using the methodology of Bruzzi et al., with adjustments for the same sets of confounders.^21^ All analyses were conducted using SAS v9.2 (SAS Institute, Inc., Cary, NC, USA).

## 3 RESULTS

This study included 2969 esophageal cancer cases and 8019 controls (Table 1). In either male or female population, cases and controls differed in the distributions of county of residence, age, education level, income 10 years ago, BMI, family history of esophageal cancer, and ever tobacco smoking (*p* < 0.001). A significant difference between cases and controls was also found in alcohol drinking in males (*p* <.001) but in females (*p* = .198). The age distribution of all cases was as follows: 4.56% for those under 50, 21.62% for ages 50–59, 34.06% for ages 60–69, and 39.76% for those 70 and older. This distribution aligned closely with the age distribution of the respondents.

**TABLE 1:** Demographic characteristics among cases and controls, by gender.

Variables	Men			Women		
	Case (*N* = 2103, %)	Control (*N* = 5767, %)	*p* ^a^	Case (*N* = 2103, %)	Control (*N* = 5767, %)	*p* ^a^
County of residence			<.001			<.001
Dafeng	427 (20.3)	1818 (31.5)		212 (24.5)	718 (31.9)	
Ganyu	806 (38.3)	1600 (27.7)		125 (14.4)	410 (18.2)	
Chouzhou	584 (27.8)	755 (13.1)		384 (44.3)	425 (18.9)	
Tongshan	286 (13.6)	1594 (27.6)		145 (16.7)	699 (31.0)	
Age (years)			<.001			<.001
<50	106 (5.0)	617 (10.7)		32 (3.7)	267 (11.9)	
50‐	493 (23.4)	1331 (23.1)		175 (20.2)	463 (20.6)	
60‐	753 (35.8)	1873 (32.5)		342 (39.5)	692 (30.7)	
≥70	751 (35.7)	1946 (33.7)		317 (36.6)	830 (36.9)	
Education level			<.001			<.001
Illiteracy	983 (46.7)	2243 (38.9)		740 (85.5)	1596 (70.9)	
Primary	801 (38.1)	2104 (36.5)		99 (11.4)	421 (18.7)	
Middle	265 (12.6)	1133 (19.6)		24 (2.8)	187 (8.3)	
High or college	54 (2.6)	287 (5.0)		3 (0.3)	48 (2.1)	
Income 10 years ago (Yuan/year)			<.001			<.001
<1000	647 (30.8)	1240 (21.5)		240 (27.7)	478 (21.2)	
1000‐	460 (21.9)	1085 (18.8)		181 (20.9)	470 (20.9)	
1500‐	553 (26.3)	1542 (26.7)		240 (27.7)	604 (26.8)	
≥2500	443 (21.1)	1900 (32.9)		205 (23.7)	700 (31.1)	
Body mass index (BMI)^b^			<.001			<.001
<18.5	291 (13.8)	290 (5.0)		177 (20.4)	165 (7.3)	
18.5–23.9	1463 (69.6)	3640 (63.1)		510 (58.9)	1235 (54.8)	
24.0–27.9	286 (13.6)	1565 (27.1)		139 (16.1)	669 (29.7)	
≥28.0	63 (3.0)	272 (4.7)		40 (4.6)	183 (8.1)	
Family history of esophagus cancer			<.001			<.001
No	1751 (83.3)	5299 (91.9)		684 (79.0)	2043 (90.7)	
Yes	352 (16.7)	468 (8.1)		182 (21.0)	209 (9.3)	
Ever smoking			<.001			<.001
No	502 (23.9)	2428 (42.1)		657 (75.9)	1864 (82.8)	
Yes	1601 (76.1)	3339 (57.9)		209 (24.1)	388 (17.2)	
Alcohol drinking			<.001			0.198
No	609 (29.0)	2358 (40.9)		763 (88.1)	1945 (86.4)	
Yes	1494 (71.0)	3409 (59.1)		103 (11.9)	307 (13.6)	

^a^ Chi‐square test for difference distribution between cases and controls.

^b^ Chinese recommended standard was used for the cutoff points of overweight and obesity.

Associations of ETS and esophageal cancer in both smokers and non‐smokers (i.e., non‐active smokers) combined are shown in Table 2. Cases were more exposed to ETS than controls, both at home and work. Nearly half of the cases (42.9%) reported exposure to ETS at home whereas 30.6% among controls. On the other hand, 15.9% of cases reported workplace exposure whereas 10.9% among controls. For ETS exposure at home, the SB‐adjusted OR for ETS exposure was 1.39 (95% CI: 1.26–1.53). When ETS was categorized into 4 levels (none, light, moderate, and heavy), the SB‐adjusted OR for heavy exposure was 1.51 (95% CI: 1.27–1.81) compared to unexposed, with a significant dose–response relationship (*P*_trend_ < 0.001). When stratified by gender, SB‐adjusted ORs for women were higher than those in men (OR = 1.61, 95% CI: 1.35–1.93 vs. OR = 1.30, 95% CI: 1.16–1.46). When further stratified by places of exposure, the OR for heavy ETS at home was 1.84 (95% CI: 1.40–2.43, *P*_trend_ <0.001) in women, which was stronger than in men with an SB‐adjusted OR of 1.33 (95% CI: 1.05–1.67, *P*_trend_ <0.001). For exposure to ETS at work, those with ETS exposure had an SB‐adjusted OR of 1.29 (95% CI: 1.13–1.48) when both men and women were combined. The association was more prominent in men (OR = 1.36, 95% CI: 1.17–1.58) and attenuated in women (OR = 0.98 95% CI: 0.71–1.35). For the level of ETS exposure at work, heavy exposure was associated with an OR of 1.82 (95% CI: 1.31–2.52, *P*_trend_ <0.001) overall, and an OR of 1.68 (95% CI: 1.17–2.41, *P*_trend_ <0.001) in men; numbers of cases and controls for women among the various levels of exposure were relatively small and produced no apparent trend.

**TABLE 2:** Associations of environmental tobacco smoke and esophageal cancer among all participants.

Variables	Case	Control	Crude OR (95%CI)	Adjusted OR (95%CI)^a^	SB‐adjusted OR (95%CI)^a^
ETS at home					
Both men and women					
No	1694 (57.1)	556 (69.4)	1.00	1.00	1.00
Yes	1275 (42.9)	2452 (30.6)	1.71 (1.57–1.86)	1.39 (1.26–1.53)	1.39 (1.26–1.53)
None	1694 (57.8)	5567 (69.8)	1.00	1.00	1.00
Light	611 (20.9)	1107 (13.9)	1.81 (1.62–2.03)	1.42 (1.25–1.60)	1.41 (1.25–1.60)
Moderate	357 (12.2)	805 (10.1)	1.46 (1.27–1.67)	1.23 (1.06–1.42)	1.22 (1.05–1.42)
Heavy	267 (9.1)	492 (6.2)	1.78 (1.52–2.09)	1.52 (1.28–1.82)	1.51 (1.27–1.81)
*P*_trend_			<0.001	<0.001	
Men					
No	1246 (59.2)	4133 (71.7)	1.00	1.00	1.00
Yes	857 (40.8)	1634 (28.3)	1.74 (1.57–1.93)	1.31 (1.16–1.47)	1.30 (1.16–1.46)
None	1246 (60.1)	4133 (72.1)	1.00	1.00	1.00
Light	457 (22.1)	808 (14.1)	1.88 (1.65–2.14)	1.34 (1.16–1.54)	1.33 (1.15–1.54)
Moderate	226 (10.9)	503 (8.8)	1.49 (1.26–1.77)	1.17 (0.97–1.41)	1.17 (0.97–1.40)
Heavy	143 (6.9)	289 (5.0)	1.64 (1.33–2.03)	1.34 (1.06–1.69)	1.33 (1.05–1.67)
*P*_trend_			<0.001	0.001	
Women					
No	448 (51.7)	1434 (63.7)	1.00	1.00	1.00
Yes	418 (48.3)	818 (36.3)	1.64 (1.40–1.92)	1.63 (1.36–1.95)	1.61 (1.35–1.93)
None	448 (52.3)	1434 (64.1)	1.00	1.00	1.00
Light	154 (18.0)	299 (13.4)	1.65 (1.32–2.06)	1.69 (1.32–2.16)	1.66 (1.31–2.11)
Moderate	131 (15.3)	302 (13.5)	1.39 (1.10–1.75)	1.40 (1.09–1.81)	1.39 (1.08–1.78)
Heavy	124 (14.5)	203 (9.1)	1.96 (1.53–2.50)	1.89 (1.43–2.50)	1.84 (1.40–2.43)
*P*_trend_			<0.001	<0.001	
ETS at work					
Both men and women					
No	2497 (84.1)	7141 (89.1)	1.00	1.00	1.00
Yes	472 (15.9)	878 (10.9)	1.54 (1.36–1.74)	1.30 (1.13–1.48)	1.29 (1.13–1.48)
None	2497 (84.2)	7141 (89.1)	1.00	1.00	1.00
Light	192 (6.5)	439 (5.5)	1.25 (1.05–1.49)	1.06 (0.87–1.28)	1.06 (0.87–1.27)
Moderate	196 (6.6)	336 (4.2)	1.67 (1.39–2.00)	1.43 (1.17–1.74)	1.42 (1.16–1.73)
Heavy	79 (2.7)	98 (1.2)	2.31 (1.71–3.11)	1.88 (1.35–2.63)	1.82 (1.31–2.52)
*P*_trend_			<0.001	<0.001	
Men					
No	1699 (80.8)	5025 (87.1)	1.00	1.00	1.00
Yes	404 (19.2)	742 (12.9)	1.61 (1.41–1.84)	1.37 (1.18–1.58)	1.36 (1.17–1.58)
None	1699 (81.0)	5025 (87.2)	1.00	1.00	1.00
Light	160 (7.6)	375 (6.5)	1.26 (1.04–1.53)	1.04 (0.84–1.29)	1.04 (0.84–1.28)
Moderate	177 (8.4)	279 (4.8)	1.88 (1.54–2.28)	1.67 (1.35–2.08)	1.65 (1.34–2.04)
Heavy	62 (3.0)	83 (1.4)	2.21 (1.58–3.09)	1.75 (1.20–2.54)	1.68 (1.17–2.41)
*P*_trend_			<0.001	<0.001	
Women					
No	798 (92.1)	2116 (94.0)	1.00	1.00	1.00
Yes	68 (7.9)	136 (6.0)	1.33 (0.98–1.79)	0.98 (0.70–1.37)	0.98 (0.71–1.35)
None	798 (92.1)	2116 (94.0)	1.00	1.00	1.00
Light	32 (3.7)	64 (2.8)	1.33 (0.86–2.04)	1.10 (0.69–1.74)	1.09 (0.70–1.69)
Moderate	19 (2.2)	57 (2.5)	0.88 (0.52–1.50)	0.55 (0.31–0.97)	0.60 (0.35–1.01)
Heavy	17 (2.0)	15 (0.7)	3.01 (1.49–6.05)	2.46 (1.10–5.52)	1.96 (0.98–3.94)
*P*_trend_			0.027	0.854	

^a^ Adjusted for sex (male = 1, female = 0, except when stratified by sex), age (continuous), education level (illiteracy = 1, primary = 2, middle = 3, high or college = 4), income 10 years ago (Yuan/year, continuous), body mass index (continuous), family history of esophagus cancer (yes = 1, no = 0), county of residence (Dafeng = 1, Ganyu = 2, Chuzhou = 3, Tongshan = 4), pack‐year of smoking (continuous), ethanol consumption (mL/week, continuous), passive smoking from home (yes = 1, no = 0, except for all variables in ETS at home), and passive smoking from work (yes = 1, no = 0, except for all variables in ETS at work).

We examined ETS at home and work exclusively among non‐smokers to mitigate potential residual confounding attributable to active tobacco consumption (Table 3). Overall, when men and women were combined, exposure to ETS at home among non‐smokers was positively associated with esophageal cancer (OR = 1.56, 95% CI: 1.34–1.83). The association among women was stronger (OR = 1.60, 95% CI: 1.31–1.96) than that among men (OR = 1.50, 95% CI: 1.17–1.93). Meanwhile, exposure to ETS at work was associated with the disease (OR = 1.25, 95% CI: 0.96–1.63), but the association was stronger among men (OR = 1.38, 95% CI: 0.97–1.96) than among women (OR = 1.12, 95% CI: 0.76–1.66). When we analyzed non‐smokers across four tiers of exposure (none, light, moderate, and heavy), exposure to ETS demonstrated a significant trend, yielding an SB‐adjusted OR of 1.47 for heavy exposure. (95% CI: 1.11–1.94, *P*_trend_ <0.001) at home and 1.92 (95% CI: 1.08–3.41, *P*_trend_ = 0.019) at work, respectively. A trend was also observed in males (*P*_trend_ = 0.012) and females (*P*_trend_ = 0.000) exposure to ETS at home, and in males (*P*_trend_ = 0.004) but not females (*P*_trend_ = 0.667) exposure to ETS at work.

**TABLE 3:** Associations of environmental tobacco smoke and esophageal cancer among non‐smokers^a^.

Variables	Case *N*, (%)	Control *N*, (%)	Crude OR (95%CI)	Adjusted OR (95%CI)^b^	SB‐adjusted OR (95%CI)^b^
ETS at home					
Both men and women					
No	737 (63.6)	3317 (77.3)	1.00	1.00	1.00
Yes	422 (36.4)	975 (22.7)	1.95 (1.70–2.24)	1.57 (1.34–1.84)	1.56 (1.34–1.83)
None	737 (64.0)	3317 (77.6)	1.00	1.00	1.00
Light	193 (16.8)	410 (9.6)	2.12 (1.75–2.56)	1.74 (1.41–2.14)	1.72 (1.40–2.11)
Moderate	126 (10.9)	324 (7.6)	1.75 (1.40–2.18)	1.43 (1.12–1.82)	1.41 (1.11–1.80)
Heavy	95 (8.3)	221 (5.2)	1.94 (1.50–2.49)	1.49 (1.12–1.99)	1.47 (1.11–1.94)
*P*_trend_			<0.001	<0.001	
Men					
No	372 (74.1)	2066 (85.1)	1.00	1.00	1.00
Yes	130 (25.9)	362 (14.9)	1.99 (1.59–2.51)	1.52 (1.18–1.97)	1.50 (1.17–1.93)
None	372 (74.5)	2066 (85.4)	1.00	1.00	1.00
Light	71 (14.2)	180 (7.4)	2.19 (1.63–2.95)	1.60 (1.16–2.21)	1.56 (1.14–2.14)
Moderate	41 (8.2)	111 (4.6)	2.05 (1.41–2.98)	1.55 (1.02–2.35)	1.50 (1.01–2.23)
Heavy	15 (3.0)	62 (2.6)	1.34 (0.76–2.39)	1.20 (0.63–2.30)	1.16 (0.64–2.09)
*P*_trend_			<0.001	0.012	
Women					
No	365 (55.6)	1251 (67.1)	1.00	1.00	1.00
Yes	292 (44.4)	613 (32.9)	1.63 (1.36–1.96)	1.62 (1.32–1.98)	1.60 (1.31–1.96)
None	365 (56.0)	1251 (67.5)	1.00	1.00	1.00
Light	122 (18.7)	230 (12.4)	1.82 (1.42–2.33)	1.88 (1.43–2.47)	1.83 (1.40–2.40)
Moderate	85 (13.0)	213 (11.5)	1.37 (1.04–1.81)	1.37 (1.01–1.85)	1.35 (1.00–1.81)
Heavy	80 (12.3)	159 (8.6)	1.73 (1.29–2.31)	1.61 (1.16–2.24)	1.57 (1.14–2.16)
*P*_trend_			<0.001	<0.001	
ETS at work					
Both men and women					
No	1060 (91.5)	4032 (93.9)	1.00	1.00	1.00
Yes	99 (8.5)	260 (6.1)	1.45 (1.14–1.84)	1.27 (0.97–1.66)	1.25 (0.96–1.63)
None	1060 (91.5)	4032 (94.0)	1.00	1.00	1.00
Light	44 (3.8)	140 (3.3)	1.20 (0.85–1.69)	1.09 (0.75–1.58)	1.08 (0.76–1.55)
Moderate	34 (2.9)	84 (2.0)	1.54 (1.03–2.31)	1.25 (0.80–1.96)	1.23 (0.80–1.88)
Heavy	21 (1.8)	33 (0.8)	2.42 (1.40–4.20)	2.20 (1.17–4.14)	1.92 (1.08–3.41)
*P*_trend_			<0.001	0.019	
Men					
No	449 (89.4)	2258 (93.0)	1.00	1.00	1.00
Yes	53 (10.6)	170 (7.0)	1.57 (1.13–2.17)	1.41 (0.98–2.02)	1.38 (0.97–1.96)
None	449 (89.4)	2258 (93.1)	1.00	1.00	1.00
Light	19 (3.8)	96 (4.0)	1.00 (0.60–1.65)	0.88 (0.52–1.51)	0.90 (0.54–1.48)
Moderate	24 (4.8)	52 (2.1)	2.32 (1.42–3.81)	2.00 (1.15–3.47)	1.82 (1.09–3.04)
Heavy	10 (2.0)	19 (0.8)	2.65 (1.22–5.73)	2.84 (1.19–6.77)	2.12 (1.01–4.42)
*P*_trend_			<0.001	0.004	
Women					
No	611 (93.0)	1774 (95.2)	1.00	1.00	1.00
Yes	46 (7.0)	90 (4.8)	1.48 (1.03–2.14)	1.13 (0.75–1.70)	1.12 (0.76–1.66)
None	611 (93.0)	1774 (95.2)	1.00	1.00	1.00
Light	25 (3.8)	44 (2.4)	1.65 (1.00–2.72)	1.40 (0.82–2.39)	1.34 (0.82–2.21)
Moderate	10 (1.5)	32 (1.7)	0.91 (0.44–1.86)	0.58 (0.27–1.25)	0.66 (0.33–1.29)
Heavy	11 (1.7)	14 (0.8)	2.28 (1.03–5.06)	1.76 (0.70–4.39)	1.48 (0.69–3.18)
*P*_trend_			0.046	0.667	

^a^ I.e., non‐active smokers.

^b^ Adjusted for sex (male = 1, female = 0, except when stratified by sex), age (continuous), education level (illiteracy = 1, primary = 2, middle = 3, high or college = 4), income 10 years ago (Yuan/year, continuous), body mass index (continuous), family history of esophagus cancer (yes = 1, no = 0), county of residence (Dafeng = 1, Ganyu = 2, Chuzhou = 3, Tongshan = 4), ethanol consumption (mL/week, continuous), passive smoking from home (yes = 1, no = 0, except for all variables in ETS at home), and passive smoking from work (yes = 1, no = 0, except for all variables in ETS at work).

We further analyzed ETS exposure when home and work were combined (Table 4). Among the overall population with both smokers and non‐smokers, a positive association was observed between ETS exposure (Yes vs. No) and esophageal cancer with an SB‐adjusted OR of 1.44 (95% CI: 1.31–1.58). Among non‐smokers, the correlation was markedly more pronounced, evidencing an SB‐adjusted OR of 1.56 (95% CI: 1.35–1.82). The PAFs were 15.0% (95% CI: 10.3%–18.9%) and 12.1% (95% CI: 8.8%–19.8%) for all and non‐smokers, respectively. For both smokers and non‐smokers combined, individuals exposed to ETS at home and work were associated with an SB‐adjusted OR of 1.79 (95% CI: 1.51–2.12) in comparison to those unexposed in either setting. Notably, exposure from a singular source (either home or work) also yielded a significant association (OR = 1.37, 95% CI: 1.24–1.51). This association was particularly stronger among non‐smokers, with an adjusted OR of 1.99 (95% CI: 1.44–2.75) for exposures from both sources and 1.50 (95% CI: 1.28–1.76) for exposure originating from any single source. The strength of the association was observed to increase with the level of combined (work and home) exposure, across varying degrees of exposure intensity (none, light, moderate, heavy). Overall, the OR for heavy exposure at home and work was 2.32 (95% CI: 1.64–3.26, *P*_trend_ <0.001). Among nonsmokers, the association for heavy ETS was 1.87 (95% CI: 1.02–3.42, *P*_trend_ <0.001).

**TABLE 4:** Combined effects of environmental tobacco smoke at home and work.

Variables	Case *N*, (%)	Control *N*, (%)	Crude OR (95%CI)	Adjusted OR (95%CI)^a^	SB‐adjusted OR (95%CI)^a^
Smokers and non‐smokers^b^					
No smoking at both home and work	1522 (51.3)	5171 (64.5)	1.00	1.00	1.00
Any exposure to smoking at home and/or work	1447 (48.7)	2848 (35.5)	1.73 (1.59–1.88)	1.44 (1.32–1.59)	1.44 (1.31–1.58)
PAF (95% CI)^1^	15.0% (10.3%–18.9%)				
Non‐smokers^b^ only					
No smoking at both home and work	704 (60.7)	3186 (74.2)	1.00	1.00	1.00
Any exposure to smoking at home and/or work	455 (39.3)	1106 (25.8)	1.86 (1.63–2.13)	1.57 (1.35–1.83)	1.56 (1.35–1.82)
PAF (95% CI)^1^	12.1% (8.8%–19.8%)				
Smokers and Non‐Smokers^b^					
No smoking at both home and work	1522 (51.3)	5171 (64.5)	1.00	1.00	1.00
Smoking at either home or work	1147 (38.6)	2366 (29.5)	1.65 (1.50–1.80)	1.38 (1.25–1.52)	1.37 (1.24–1.51)
Smoking at both home and work	300 (10.1)	482 (6.0)	2.12 (1.81–2.47)	1.80 (1.52–2.14)	1.79 (1.51–2.12)
*P*_trend_			<0.001	<0.001	
Non‐smokers^b^ only					
No smoking at both home and work	704 (60.7)	3186 (74.2)	1.00	1.00	1.00
Smoking at either home or work	389 (33.6)	977 (22.8)	1.80 (1.56–2.08)	1.51 (1.29–1.77)	1.50 (1.28–1.76)
Smoking at both home and work	66 (5.7)	129 (3.0)	2.32 (1.70–3.15)	2.07 (1.48–2.89)	1.99 (1.44–2.75)
*P*_trend_			<0.001	<0.001	
Smokers and non‐smokers^b^					
None	1522 (52.0)	5171 (64.9)	1.00	1.00	1.00
Light	991 (33.9)	2019 (25.3)	1.67 (1.52–1.83)	1.36 (1.23–1.51)	1.36 (1.23–1.51)
Moderate	341 (11.7)	693 (8.7)	1.67 (1.45–1.93)	1.53 (1.31–1.79)	1.52 (1.30–1.78)
Heavy	73 (2.5)	83 (1.0)	2.99 (2.17–4.11)	2.45 (1.72–3.48)	2.32 (1.64–3.26)
*P*_trend_			<0.001	<0.001	
Non‐smokers^b^ only					
None	704 (61.2)	3186 (74.6)	1.00	1.00	1.00
Light	316 (27.5)	788 (18.5)	1.82 (1.56–2.12)	1.53 (1.29–1.81)	1.52 (1.29–1.80)
Moderate	114 (9.9)	264 (6.2)	1.95 (1.55–2.47)	1.69 (1.30–2.19)	1.66 (1.28–2.14)
Heavy	17 (1.5)	31 (0.7)	2.48 (1.37–4.51)	2.16 (1.10–4.25)	1.87 (1.02–3.42)
*P*_trend_			<0.001	<0.001	

^a^ Adjusted for sex (male = 1, female = 0), age (continuous), education level (illiteracy = 1, primary = 2, middle = 3, high or college = 4), income 10 years ago (Yuan/year, continuous), body mass index (continuous), family history of esophagus cancer (yes = 1, no = 0), county of residence (Dafeng = 1, Ganyu = 2, Chuzhou = 3, Tongshan = 4), pack‐year of smoking (continuous, except for only nonsmokers [i.e., non‐active smokers]), and ethanol consumption (mL/week, continuous).

^b^ I.e., non‐active smokers.

Besides exposure degree, we further analyzed exposure duration and exposure intensity. Similarly, individuals exposed to ETS, using degree, intensity, or duration as proxies, generally faced a higher risk of EC compared to those who were not exposed (Supplementary Table S1–S5). The results were generally consistent with associations based on ETS exposure degree, however, the dose–response relationships were not observed in ETS at work among non‐smokers, when using cumulative intensity, especially using duration of exposure.

## 4 DISCUSSION

In this case–control study, we observed that esophageal cancer is associated with exposure to ETS whether at home, at work, or in both environments. The associations were evident among both smokers and non‐smokers combined and among nonsmokers with dose–response relationships. The positive associations were consistent in stratified analysis by gender, except ETS at work among women. We found that about 15.0% and 12.1% of cases could be attributable to ETS exposure at both home and work for the overall population and nonsmokers, respectively. These findings are critical given the absence of a comprehensive smoke‐free law at the national level in China. Despite the continuous efforts of tobacco control in China for several decades, second‐hand smoke remains a prevalent problem in indoor occupational settings, and the majority of workplaces are not covered by smoke‐free regulations. Our results offer some support for shaping workplace policies, particularly considering the impact of ETS among non‐smokers.

Despite limited focus on ETS's impact on esophageal cancer, its association with lung cancer in non‐smokers has been widely documented. Hackshaw et al. conducted a meta‐analysis of 39 studies to assess the risk of lung cancer among non‐smokers with smoking spouses and estimated a pooled OR of 1.23 (95% CI: 1.13–1.34) across both genders.^22^ Our findings of ETS‐esophageal cancer among non‐smokers align with the conclusions drawn in the ETS‐lung cancer meta‐analysis. Particularly, the point estimates for ETS‐associated esophageal cancer among the Chinese population suggest similar biological pathways may underlie the carcinogenesis of both lung and esophageal cancers.

A few papers have explored the relationship between ETS and esophageal cancer. A case–control study from Huai'an in Jiangsu Province, China, examined 107 cases of squamous cell esophageal cancer and reported a crude OR of 2.04 (95% CI: 1.14–3.70). Nonetheless, this link was not observed in the multivariate analysis upon adjustment for potential confounders.^18^ Another Chinese case–control study examining 250 cases of squamous cell esophageal cancer identified an adjusted OR of 2.42 (95% CI: 1.36–4.32), but this analysis did not account for active smoking as a confounding factor in its multivariate approach.^17^ The two studies on Chinese populations faced methodological limitations, especially the relatively small sample sizes and a lack of sufficient statistical power to conduct further analyses specifically among non‐smokers. Other than the Chinese population, a study from the Unite States evaluated the impact of ETS on esophageal adenocarcinoma and observed an OR of 1.49 (95% CI: 0.65–3.40) for non‐smokers exposed to ETS, although the analysis was based on only 22 non‐smoking cases.^23^ Secondhand smoke was also identified as a risk factor for the early onset among younger patients in Tanzania.^24^ A recent hospital‐based case–control study among 703 esophageal squamous cell carcinoma cases and 1664 matched controls in India found that every exposure to ETS and exposure to ETS for >14 h/week were positively associated with squamous cell esophageal cancer risk both among all participants and non‐smokers. However, the associations were driven toward the null after adjusting for confounders including active smoking. The study was still limited by sample size since only a total of 61 cases and 31 non‐smoker cases were ever exposed to ETS.^25^ Passive smoking was also suggested to have an impact earlier in the progression of the disease with an impact on precancerous lesions.^26^ This case–control study, to our best understanding, includes the largest sample size with observed positive correlations between ETS exposure and esophageal cancer. Additionally, it is the first to investigate the impact of ETS on esophageal cancer in non‐smokers among Chinese.

Involuntary smoking (or ETS) has been classified as a Group I carcinogen with substantial evidence supporting its association with lung cancer.^22^ Nonetheless, the linkage between ETS and other cancers related to smoking remains relatively understudied. IARC has identified at least 69 carcinogenic compounds within ETS (e.g., polycyclic aromatic hydrocarbons, tobacco‐specific N‐nitrosamines) that possess the capability to inflict direct harm on various tissues, such as the esophagus.^27^ It is traditionally believed that ETS can induce genetic mutations of the epithelium of target tissues.^28^, ^29^ ETS exposure in non‐smokers is linked to higher levels of 4‐ABP‐hemoglobin adducts.^30^ Recent findings suggest that its carcinogenic effects are enhanced by its ability to accelerate the premature aging of mitochondria via oxidative mitochondrial metabolism.^31^

Considering the biological plausibility and substantial empirical support from studies on lung cancer, the observed correlation between ETS and esophageal cancer discerned in our large‐scale study is unlikely to be coincidental. Our evaluation accounted for possible confounders by combining smokers and non‐smokers, specifically addressing active tobacco usage and alcohol consumption, which are predominant esophageal cancer risk factors among the Chinese. Moreover, to diminish residual confounding of active smoking, we performed analyses exclusively among non‐smokers. Since active smoking is a known variable for EC and is likely to have a confounding effect in the analysis of ETS and EC, one way to avoid the confounding is to observe the impact on the non‐smoking population. In our results of combined analyses presented in Table 4, we observed point estimates of ETS‐disease were strengthened for non‐smokers only compared to that for the overall population with both smokers and non‐smokers.

The retrospective design of the case–control study and the reliance on self‐reported exposures could introduce differential recall bias among cases and controls, potentially resulting in an overestimated association between ETS and esophageal cancer. However, given that ETS has not been previously identified as a risk factor for esophageal cancer in the Chinese population, the concern regarding potential differential recall bias may be mitigated. Additionally, self‐reported ETS has been suggested to be a valid measure for exposure.^32^ Despite being a population‐based case–control study, the potential for selection bias cannot be entirely ignored due to the short survival after diagnosis of the disease. Consequently, patients in advanced stages may die before we approach and recruit them into the study. This could partially account for the relatively lower response rate of 30% among cases. Given the potential correlation between smoking and stages of cancer, the observed association could potentially be underestimated. Moreover, while we acknowledge the importance of subtypes, we were unable to do so due to a substantial proportion of missing data (over 55%). For those with recorded subtypes, 33.6% are adenocarcinoma and 66.4% are squamous cell carcinoma. It has been recently reported based on China's national data, that approximately 94.2% of esophageal cancer cases in China are esophageal squamous cell carcinoma.^33^ Lastly, the data collection process for the duration of ETS exposure was challenging and might affect precision, potentially suffering from selection bias due to missing data, which could lead to inconsistent dose–response association. Although lifetime exposure to ETS was collected in this study, due to the nature of a case–control study design, there is a possibility of temporal ambiguity, which might distort the observed association. Despite the aforementioned limitations, the primary strength of this study is its large, population‐based case–control design conducted in a region with high incidence rates of esophageal cancer. Epidemiologic data were collected systematically using a comprehensive questionnaire with quality control procedures.

In conclusion, the observed associations suggest exposure to ETS could be a critical factor in the development of esophageal cancer. Given that China continues to be a major consumer of tobacco products, with approximately 53.3% of adult males smoking, various intervention programs have been implemented to reduce tobacco‐related diseases, including cancer.^34^, ^35^ Ideally, reducing overall smoking rates would be the goal. However, in light of these findings, it may be beneficial to target ETS intervention programs in the workplace and at home. Our estimates for population‐attributable fractions further indicate that effective interventions against ETS exposure, both in the workplace and residential environments, could substantially lower the incidence of esophageal cancer in China as well as across other parts of the world.

## AUTHOR CONTRIBUTIONS

**Zi‐Yi Jin:** Formal analysis; visualization; writing – original draft. **Kuangyu Liu:** Formal analysis; visualization; writing – original draft. **Gina Wallar:** Formal analysis; writing – original draft. **Jin‐Yi Zhou:** Data curation; investigation; software; supervision; writing – review and editing. **Li‐Na Mu:** Data curation; investigation; supervision; writing – review and editing. **Xing Liu:** Data curation; formal analysis; validation; writing – review and editing. **Li‐Ming Li:** Conceptualization; methodology; resources; validation; writing – review and editing. **Na He:** Conceptualization; methodology; resources; validation; writing – review and editing. **Ming Wu:** Conceptualization; data curation; funding acquisition; investigation; methodology; project administration; resources; software; supervision; writing – review and editing. **Jin‐Kou Zhao:** Conceptualization; funding acquisition; investigation; methodology; project administration; resources; software; supervision; writing – review and editing. **Zuo‐Feng Zhang:** Conceptualization; funding acquisition; investigation; methodology; project administration; resources; software; supervision; writing – review and editing.

## FUNDING INFORMATION

This project was funded by the Jiangsu Provincial Health Department (RC 2003090); the National Institutes of Health, National Institute of Environmental Health Sciences, National Cancer Institute, Department of Health and Human Services (ES06718, ES011667, CA90833, CA077954, CA96134, DA11386, and CA009142).

## CONFLICT OF INTEREST STATEMENT

All authors declare they have no conflict of interests.

## ETHICS STATEMENT

Every participant provided written informed consent. The study protocol was approved by the Institutional Review Board of the Jiangsu Provincial Health Department.

## Supporting information


**Data S1.** Supporting Information.

## Data Availability

The data supporting the findings of this study are available upon request from the corresponding author.
