# Supplementary material for: Environmental tobacco smoking (ETS) and esophageal cancer: A population‐based case‐control study in Jiangsu Province, China
Source: Int J Cancer. 2024 Nov 18;156(8):1552–62. doi: 10.1002/ijc.35254 (PMC11826109; doi:10.1002/ijc.35254)
Supplement: Supplementary file 1 — Data S1. Supporting Information. [file IJC-156-1552-s001.pdf]

Environmental Tobacco Smoking (ETS) and Esophageal Cancer: A Population-Based Case-Control Study in Jiangsu Province, China

Authors:

Zi-Yi Jin, Kuangyu Liu, Gina Wallar, Jin-Yi Zhou, Li-Na Mu, Xing Liu, Li-Ming Li, Na He, Ming Wu, Jin-Kou Zhao, Zuo-Feng Zhang

Supplementary material

Contents

Table S1. Associations of environmental tobacco smoke and esophageal cancer among all participants using the exposure duration as the exposure (i.e., exposure years in lifetime).

Table S2. Associations of environmental tobacco smoke and esophageal cancer among all participants using the lifetime exposure intensity as the exposure. The lifetime exposure intensity was calculated by multiplying the exposure degree by the exposure duration and adding the exposure from different family members or different workplaces. The lifetime exposure intensity was divided into five groups, including no exposure to ETS, and other four groups by the quartile of the exposure intensity to ETS in the control group.

Table S3. Associations of environmental tobacco smoke and esophageal cancer among non-smokers using the exposure duration as the exposure (i.e., exposure years in lifetime).

Table S4. Associations of environmental tobacco smoke and esophageal cancer among non-smokers using the lifetime exposure intensity as the exposure. The lifetime exposure intensity was calculated by multiplying the exposure degree by the exposure duration and adding the exposure from different family members or different workplaces. The lifetime exposure intensity was divided into five groups, including no exposure to ETS, and other four groups by the quartile of the exposure intensity to ETS in the control group.

Table S5. Combined effects of environmental tobacco smoke at home and at work using the lifetime exposure intensity as the exposure. The lifetime exposure intensity was calculated by multiplying the exposure degree by the exposure duration and adding the exposure from different family members or different workplaces. The lifetime exposure intensity was divided into five groups, including no exposure to ETS, and other four groups by the quartile of the exposure intensity to ETS in the control group.

Table S1. Associations of environmental tobacco smoke and esophageal cancer among all participants using the exposure duration as the exposure (i.e., exposure years in lifetime).

Variables	Case N (%)	Control N (%)	Crude OR(95%CI)	Adjusted OR(95%CI)[a]	SB-Adjusted OR(95%CI)[a]
**ETS at home**					
**Both men and women**					
No	1,694(57.1)	5,567(69.4)	1.00	1.00	1.00
Yes	1,275(42.9)	2,452(30.6)	1.71(1.57-1.86)	1.39(1.26-1.53)	1.39(1.26-1.53)
None	1,694(57.8)	5,567(69.8)	1.00	1.00	1.00
Quartile 1	329(11.2)	597(7.5)	1.81(1.57-2.09)	1.43(1.22-1.67)	1.42(1.21-1.66)
Quartile 2	244(8.3)	564(7.1)	1.42(1.21-1.67)	1.22(1.03-1.45)	1.22(1.02-1.45)
Quartile 3	323(11.0)	575(7.2)	1.85(1.59-2.14)	1.55(1.32-1.82)	1.54(1.31-1.80)
Quartile 4	339(11.6)	668(8.4)	1.67(1.45-1.92)	1.31(1.12-1.53)	1.30(1.12-1.52)
P_{trend}			<0.001	<0.001	
**Men**					
No	1,246(59.2)	4,133(71.7)	1.00	1.00	1.00
Yes	857(40.8)	1,634(28.3)	1.74(1.57-1.93)	1.31(1.16-1.47)	1.30(1.16-1.46)
None	1,246(60.1)	4,133(72.1)	1.00	1.00	1.00
Quartile 1	145(7.0)	309(5.4)	1.56(1.27-1.92)	1.11(0.89-1.39)	1.11(0.89-1.39)
Quartile 2	257(12.4)	426(7.4)	2.00(1.69-2.37)	1.48(1.23-1.78)	1.47(1.23-1.77)
Quartile 3	223(10.8)	463(8.1)	1.60(1.35-1.90)	1.27(1.05-1.54)	1.27(1.05-1.53)
Quartile 4	201(9.7)	402(7.0)	1.66(1.38-1.99)	1.24(1.02-1.51)	1.23(1.01-1.50)
P_{trend}			<0.001	<0.001	
**Women**					
No	448(51.7)	1,434(63.7)	1.00	1.00	1.00
Yes	418(48.3)	818(36.3)	1.64(1.40-1.92)	1.63(1.36-1.95)	1.61(1.35-1.93)
None	448(52.3)	1,434(64.1)	1.00	1.00	1.00
Quartile 1	67(7.8)	154(6.9)	1.39(1.03-1.89)	1.74(0.97-3.12)	1.60(0.93-2.74)
Quartile 2	113(13.2)	247(11.0)	1.46(1.14-1.87)	0.53(0.12-2.43)	0.75(0.27-2.09)
Quartile 3	116(13.5)	200(8.9)	1.86(1.44-2.39)	1.07(0.67-1.71)	1.06(0.68-1.66)
Quartile 4	113(13.2)	203(9.1)	1.78(1.38-2.30)	1.67(0.97-2.86)	1.56(0.94-2.58)
P_{trend}			<0.001	<0.001	
**ETS at work**					
**Both men and women**					
No	2,497(84.1)	7,141(89.1)	1.00	1.00	1.00
Yes	472(15.9)	878(10.9)	1.54(1.36-1.74)	1.30(1.13-1.48)	1.29(1.13-1.48)
None	2,497(84.2)	7,141(89.1)	1.00	1.00	1.00
Quartile 1	53(1.8)	111(1.4)	1.37(0.98-1.90)	1.28(0.90-1.83)	1.26(0.89-1.78)
Quartile 2	117(3.9)	205(2.6)	1.63(1.30-2.06)	1.52(1.18-1.95)	1.50(1.17-1.92)
Quartile 3	208(7.0)	334(4.2)	1.78(1.49-2.13)	1.59(1.31-1.94)	1.58(1.30-1.92)

Quartile 4	89(3.0)	223(2.8)	1.14(0.89-1.47)	0.75(0.57-0.98)	0.75(0.58-0.98)
P_{trend}			<0.001	0.016	
**Men**					
No	1,699(80.8)	5,025(87.1)	1.00	1.00	1.00
Yes	404(19.2)	742(12.9)	1.61(1.41-1.84)	1.37(1.18-1.58)	1.36(1.17-1.58)
None	1,699(81.0)	5,025(87.2)	1.00	1.00	1.00
Quartile 1	46(2.2)	100(1.7)	1.36(0.96-1.94)	1.27(0.87-1.85)	1.25(0.87-1.80)
Quartile 2	103(4.9)	177(3.1)	1.72(1.34-2.21)	1.57(1.19-2.06)	1.54(1.18-2.01)
Quartile 3	185(8.8)	274(4.8)	2.00(1.65-2.43)	1.80(1.45-2.23)	1.77(1.43-2.19)
Quartile 4	65(3.1)	186(3.2)	1.03(0.78-1.38)	0.69(0.51-0.94)	0.70(0.52-0.95)
P_{trend}			<0.001	0.006	
**Women**					
No	798(92.1)	2,116(94.0)	1.00	1.00	1.00
Yes	68(7.9)	136(6.0)	1.33(0.98-1.79)	0.98(0.70-1.37)	0.98(0.71-1.35)
None	798(92.1)	2,116(94.0)	1.00	1.00	1.00
Quartile 1	15(1.7)	23(1.0)	1.49(1.06-2.08)	1.66(0.87-3.15)	1.51(0.85-2.71)
Quartile 2	17(2.0)	44(2.0)	1.57(1.20-2.07)	0.31(0.06-1.55)	0.61(0.21-1.74)
Quartile 3	12(1.4)	29(1.3)	1.76(1.34-2.33)	0.82(0.49-1.36)	0.84(0.52-1.35)
Quartile 4	24(2.8)	40(1.8)	1.66(1.25-2.19)	1.00(0.55-1.80)	1.00(0.58-1.72)
P_{trend}			0.102	0.600	

[a] Adjusted for sex (male = 1, female = 0, except when stratified by sex), age (continuous), education level (illiteracy = 1, primary = 2, middle = 3, high or college = 4), income 10 years ago (Yuan/year, continuous), body mass index (continuous), family history of esophagus cancer (yes = 1, no = 0), county of residence (Dafeng = 1, Ganyu = 2, Chuzhou = 3, Tongshan = 4), pack-year of smoking (continuous), ethanol consumption (ml/week, continuous), passive smoking from home (yes = 1, no = 0, except for all variables in ETS at home), and passive smoking from work (yes = 1, no = 0, except for all variables in ETS at work).

Table S2. Associations of environmental tobacco smoke and esophageal cancer among all participants using the lifetime exposure intensity as the exposure. The lifetime exposure intensity was calculated by multiplying the exposure degree by the exposure duration and adding the exposure from different family members or different workplaces. The lifetime exposure intensity was divided into five groups, including no exposure to ETS, and other four groups by the quartile of the exposure intensity to ETS in the control group.

Variables	Case N (%)	Control N (%)	Crude OR(95%CI)	Adjusted OR(95%CI)[a]	SB-Adjusted OR(95%CI)[a]
**ETS at home**					
			**Both men and women**		
No	1,694(57.1)	5,567(69.4)	1.00	1.00	1.00
Yes	1,275(42.9)	2,452(30.6)	1.71(1.57-1.86)	1.39(1.26-1.53)	1.39(1.26-1.53)
None	1,694(57.1)	5,567(69.4)	1.00	1.00	1.00
Quartile 1	316(10.6)	597(7.4)	1.74(1.50-2.02)	1.42(1.21-1.67)	1.41(1.20-1.66)
Quartile 2	290(9.8)	615(7.7)	1.55(1.33-1.80)	1.26(1.07-1.49)	1.26(1.07-1.48)
Quartile 3	294(9.9)	587(7.3)	1.65(1.42-1.91)	1.39(1.18-1.64)	1.39(1.18-1.63)
Quartile 4	375(12.6)	653(8.1)	1.89(1.64-2.17)	1.49(1.27-1.73)	1.48(1.27-1.72)
P_{trend}			<0.001	<0.001	
			**Men**		
No	1,246(59.2)	4,133(71.7)	1.00	1.00	1.00
Yes	857(40.8)	1,634(28.3)	1.74(1.57-1.93)	1.31(1.16-1.47)	1.30(1.16-1.46)
None	1,246(59.2)	4,133(71.7)	1.00	1.00	1.00
Quartile 1	195(9.3)	362(6.3)	1.79(1.49-2.15)	1.31(1.07-1.60)	1.30(1.06-1.58)
Quartile 2	195(9.3)	350(6.1)	1.85(1.53-2.23)	1.37(1.12-1.68)	1.36(1.11-1.66)
Quartile 3	249(11.8)	480(8.3)	1.72(1.46-2.03)	1.37(1.15-1.64)	1.36(1.14-1.63)
Quartile 4	218(10.4)	442(7.7)	1.64(1.38-1.95)	1.19(0.98-1.45)	1.19(0.98-1.44)
P_{trend}			<0.001	<0.001	
			**Women**		
No	448(51.7)	1,434(63.7)	1.00	1.00	1.00
Yes	418(48.3)	818(36.3)	1.64(1.40-1.92)	1.63(1.36-1.95)	1.61(1.35-1.93)
None	448(51.7)	1,434(63.7)	1.00	1.00	1.00
Quartile 1	72(8.3)	187(8.3)	1.23(0.92-1.65)	1.33(0.97-1.83)	1.31(0.96-1.78)
Quartile 2	90(10.4)	219(9.7)	1.32(1.01-1.72)	1.38(1.04-1.85)	1.36(1.03-1.81)
Quartile 3	124(14.3)	207(9.2)	1.92(1.50-2.45)	1.90(1.45-2.49)	1.86(1.42-2.42)
Quartile 4	132(15.2)	205(9.1)	2.06(1.62-2.63)	1.86(1.42-2.43)	1.81(1.39-2.36)
P_{trend}			<0.001	<0.001	
**ETS at work**					
			**Both men and women**		
No	2,497(84.1)	7,141(89.1)	1.00	1.00	1.00
Yes	472(15.9)	878(10.9)	1.54(1.36-1.74)	1.30(1.13-1.48)	1.29(1.13-1.48)

None	2,497(84.1)	7,141(89.1)	1.00	1.00	1.00
Quartile 1	98(3.3)	211(2.6)	1.33(1.04-1.70)	1.25(0.96-1.63)	1.24(0.96-1.61)
Quartile 2	89(3.0)	144(1.8)	1.77(1.35-2.31)	1.69(1.27-2.26)	1.65(1.25-2.19)
Quartile 3	137(4.6)	282(3.5)	1.39(1.13-1.71)	1.13(0.90-1.42)	1.13(0.90-1.41)
Quartile 4	148(5.0)	241(3.0)	1.76(1.42-2.17)	1.32(1.05-1.66)	1.31(1.04-1.64)
P_{trend}			<0.001	0.001	
			**Men**		
No	1,699(80.8)	5,025(87.1)	1.00	1.00	1.00
Yes	404(19.2)	742(12.9)	1.61(1.41-1.84)	1.37(1.18-1.58)	1.36(1.17-1.58)
None	1,699(80.8)	5,025(87.1)	1.00	1.00	1.00
Quartile 1	80(3.8)	179(3.1)	1.32(1.01-1.73)	1.28(0.95-1.71)	1.26(0.95-1.68)
Quartile 2	81(3.9)	127(2.2)	1.89(1.42-2.51)	1.71(1.26-2.33)	1.67(1.23-2.25)
Quartile 3	119(5.7)	235(4.1)	1.50(1.19-1.88)	1.18(0.93-1.52)	1.18(0.92-1.50)
Quartile 4	124(5.9)	201(3.5)	1.83(1.45-2.30)	1.45(1.12-1.87)	1.43(1.11-1.84)
P_{trend}			<0.001	<0.001	
			**Women**		
No	798(92.1)	2,116(94.0)	1.00	1.00	1.00
Yes	68(7.9)	136(6.0)	1.33(0.98-1.79)	0.98(0.70-1.37)	0.98(0.71-1.35)
None	798(92.1)	2,116(94.0)	1.00	1.00	1.00
Quartile 1	15(1.7)	23(1.0)	1.73(0.90-3.33)	1.37(0.67-2.82)	1.28(0.68-2.43)
Quartile 2	17(2.0)	44(2.0)	1.02(0.58-1.80)	0.90(0.49-1.65)	0.92(0.53-1.59)
Quartile 3	12(1.4)	29(1.3)	1.10(0.56-2.16)	0.94(0.45-1.95)	0.95(0.50-1.82)
Quartile 4	24(2.8)	40(1.8)	1.59(0.95-2.66)	0.88(0.50-1.55)	0.90(0.53-1.51)
P_{trend}			0.080	0.662	

[a] Adjusted for sex (male = 1, female = 0, except when stratified by sex), age (continuous), education level (illiteracy = 1, primary = 2, middle = 3, high or college = 4), income 10 years ago (Yuan/year, continuous), body mass index (continuous), family history of esophagus cancer (yes = 1, no = 0), county of residence (Dafeng = 1, Ganyu = 2, Chuzhou = 3, Tongshan = 4), pack-year of smoking (continuous), ethanol consumption (ml/week, continuous), passive smoking from home (yes = 1, no = 0, except for all variables in ETS at home), and passive smoking from work (yes = 1, no = 0, except for all variables in ETS at work).

Table S3. Associations of environmental tobacco smoke and esophageal cancer among non-smokers [a] using the exposure duration as the exposure (i.e., exposure years in lifetime).

Variables	Case N (%)	Control N (%)	Crude OR(95%CI)	Adjusted OR(95%CI)[b]	SB-Adjusted OR(95%CI)[b]
**ETS at home**					
**Both men and women**					
No	737(63.6)	3,317(77.3)	1.00	1.00	1.00
Yes	422(36.4)	975(22.7)	1.95(1.70-2.24)	1.57(1.34-1.84)	1.56(1.34-1.83)
None	737(64.0)	3,317(77.6)	1.00	1.00	1.00
Quartile 1	83(7.2)	208(4.9)	1.80(1.38-2.35)	1.61(1.20-2.16)	1.54(1.15-2.06)
Quartile 2	84(7.3)	267(6.3)	1.42(1.09-1.83)	1.35(1.02-1.80)	1.44(1.12-1.85)
Quartile 3	101(8.8)	186(4.4)	2.44(1.89-3.15)	1.82(1.37-2.41)	1.48(1.14-1.92)
Quartile 4	146(12.7)	294(6.9)	2.24(1.81-2.77)	1.59(1.25-2.01)	1.74(1.36-2.23)
P_{trend}			<0.001	<0.001	
**Men**					
No	372(74.1)	2,066(85.1)	1.00	1.00	1.00
Yes	130(25.9)	362(14.9)	1.99(1.59-2.51)	1.52(1.18-1.97)	1.50(1.17-1.93)
None	372(74.5)	2,066(85.4)	1.00	1.00	1.00
Quartile 1	31(6.2)	85(3.5)	2.03(1.32-3.10)	1.55(0.97-2.47)	1.48(0.92-2.36)
Quartile 2	25(5.0)	77(3.2)	1.80(1.13-2.87)	1.54(0.93-2.57)	1.30(0.80-2.09)
Quartile 3	22(4.4)	96(4.0)	1.27(0.79-2.05)	1.07(0.64-1.78)	1.40(0.95-2.07)
Quartile 4	49(9.8)	95(3.9)	2.87(2.00-4.11)	1.89(1.27-2.83)	1.68(1.10-2.54)
P_{trend}			<0.001	0.002	
**Women**					
No	365(55.6)	1,251(67.1)	1.00	1.00	1.00
Yes	292(44.4)	613(32.9)	1.63(1.36-1.96)	1.62(1.32-1.98)	1.60(1.31-1.96)
None	365(56.0)	1,251(67.5)	1.00	1.00	1.00
Quartile 1	44(6.7)	102(5.5)	1.48(1.02-2.15)	1.63(1.08-2.44)	1.38(0.97-1.97)
Quartile 2	86(13.2)	199(10.7)	1.48(1.12-1.96)	1.56(1.14-2.12)	1.45(1.05-1.99)
Quartile 3	82(12.6)	141(7.6)	1.99(1.48-2.68)	1.86(1.34-2.57)	1.66(1.21-2.28)
Quartile 4	75(11.5)	160(8.6)	1.61(1.19-2.16)	1.51(1.09-2.08)	1.78(1.31-2.41)
P_{trend}			<0.001	<0.001	
**ETS at work**					
**Both men and women**					
No	1,060(91.5)	4,032(93.9)	1.00	1.00	1.00
Yes	99(8.5)	260(6.1)	1.45(1.14-1.84)	1.27(0.97-1.66)	1.25(0.96-1.63)
None	1,060(91.5)	4,032(94.0)	1.00	1.00	1.00
Quartile 1	27(2.3)	63(1.5)	1.63(1.03-2.57)	1.88(1.15-3.09)	1.46(0.88-2.41)
Quartile 2	9(0.8)	38(0.9)	0.90(0.43-1.87)	0.95(0.43-2.11)	0.88(0.50-1.57)
Quartile 3	23(2.0)	72(1.7)	1.22(0.76-1.95)	1.26(0.76-2.11)	1.09(0.68-1.74)

Quartile 4	40(3.5)	84(2.0)	1.81(1.24-2.66)	1.08(0.71-1.63)	1.48(0.96-2.27)
P_{trend}			0.002	0.337	
**Men**					
No	449(89.4)	2,258(93.0)	1.00	1.00	1.00
Yes	53(10.6)	170(7.0)	1.57(1.13-2.17)	1.41(0.98-2.02)	1.38(0.97-1.96)
None	449(89.4)	2,258(93.1)	1.00	1.00	1.00
Quartile 1	7(1.4)	21(0.9)	1.68(0.71-3.97)	1.39(0.55-3.51)	1.29(0.65-2.56)
Quartile 2	12(2.4)	56(2.3)	1.08(0.57-2.03)	1.27(0.64-2.50)	0.80(0.40-1.60)
Quartile 3	14(2.8)	44(1.8)	1.60(0.87-2.95)	1.52(0.78-2.99)	1.34(0.72-2.47)
Quartile 4	20(4.0)	46(1.9)	2.19(1.28-3.73)	1.50(0.83-2.70)	1.86(1.09-3.17)
P_{trend}			0.002	0.058	
**Women**					
No	611(93.0)	1,774(95.2)	1.00	1.00	1.00
Yes	46(7.0)	90(4.8)	1.48(1.03-2.14)	1.13(0.75-1.70)	1.12(0.76-1.66)
None	611(93.0)	1,774(95.2)	1.00	1.00	1.00
Quartile 1	16(2.4)	22(1.2)	2.11(1.10-4.05)	1.94(0.95-3.94)	1.84(0.88-3.84)
Quartile 2	1(0.2)	2(0.1)	1.45(0.13-16.04)	1.23(0.06-27.14)	0.99(0.53-1.84)
Quartile 3	16(2.4)	42(2.3)	1.11(0.62-1.98)	0.91(0.49-1.70)	0.89(0.40-1.95)
Quartile 4	13(2.0)	24(1.3)	1.57(0.80-3.11)	0.89(0.43-1.87)	0.98(0.52-1.85)
P_{trend}			0.124	0.883	

[a] i.e., non-active smokers. [b] Adjusted for sex (male = 1, female = 0, except when stratified by sex), age (continuous), education level (illiteracy = 1, primary = 2, middle = 3, high or college = 4), income 10 years ago (Yuan/year, continuous), body mass index (continuous), family history of esophagus cancer (yes = 1, no = 0), county of residence (Dafeng = 1, Ganyu = 2, Chuzhou = 3, Tongshan = 4), ethanol consumption (ml/week, continuous), passive smoking from home (yes = 1, no = 0, except for all variables in ETS at home), and passive smoking from work (yes = 1, no = 0, except for all variables in ETS at work).

Table S4. Associations of environmental tobacco smoke and esophageal cancer among non-smokers [a] using the lifetime exposure intensity as the exposure. The lifetime exposure intensity was calculated by multiplying the exposure degree by the exposure duration and adding the exposure from different family members or different workplaces. The lifetime exposure intensity was divided into five groups, including no exposure to ETS, and other four groups by the quartile of the exposure intensity to ETS in the control group.

Variables	Case N (%)	Control N (%)	Crude OR(95%CI)	Adjusted OR(95%CI)[b]	SB-Adjusted OR(95%CI)[b]
**ETS at home**					
			**Both men and women**		
No	737(63.6)	3,317(77.3)	1.00	1.00	1.00
Yes	422(36.4)	975(22.7)	1.95(1.70-2.24)	1.57(1.34-1.84)	1.56(1.34-1.83)
None	737(63.6)	3,317(77.3)	1.00	1.00	1.00
Quartile 1	75(6.5)	217(5.1)	1.56(1.18-2.05)	1.57(1.17-2.12)	1.54(1.15-2.06)
Quartile 2	108(9.3)	269(6.3)	1.81(1.43-2.29)	1.46(1.13-1.88)	1.44(1.12-1.85)
Quartile 3	106(9.1)	242(5.6)	1.97(1.55-2.51)	1.50(1.15-1.96)	1.48(1.14-1.92)
Quartile 4	133(11.5)	247(5.8)	2.42(1.93-3.04)	1.77(1.38-2.28)	1.74(1.36-2.23)
P_{trend}			<0.001	<0.001	
			**Men**		
No	372(74.1)	2,066(85.1)	1.00	1.00	1.00
Yes	130(25.9)	362(14.9)	1.99(1.59-2.51)	1.52(1.18-1.97)	1.50(1.17-1.93)
None	372(74.1)	2,066(85.1)	1.00	1.00	1.00
Quartile 1	26(5.2)	72(3.0)	2.01(1.26-3.18)	1.55(0.94-2.56)	1.48(0.92-2.36)
Quartile 2	23(4.6)	83(3.4)	1.54(0.96-2.47)	1.34(0.81-2.24)	1.30(0.80-2.09)
Quartile 3	43(8.6)	116(4.8)	2.06(1.43-2.97)	1.44(0.96-2.17)	1.40(0.95-2.07)
Quartile 4	38(7.6)	91(3.7)	2.32(1.56-3.44)	1.76(1.14-2.74)	1.68(1.10-2.54)
P_{trend}			<0.001	0.002	
			**Women**		
No	365(55.6)	1,251(67.1)	1.00	1.00	1.00
Yes	292(44.4)	613(32.9)	1.63(1.36-1.96)	1.62(1.32-1.98)	1.60(1.31-1.96)
None	365(55.6)	1,251(67.1)	1.00	1.00	1.00
Quartile 1	54(8.2)	144(7.7)	1.29(0.92-1.80)	1.42(0.98-2.04)	1.38(0.97-1.97)
Quartile 2	69(10.5)	162(8.7)	1.46(1.08-1.98)	1.48(1.06-2.05)	1.45(1.05-1.99)
Quartile 3	79(12.0)	153(8.2)	1.77(1.32-2.38)	1.71(1.23-2.37)	1.66(1.21-2.28)
Quartile 4	90(13.7)	154(8.3)	2.00(1.51-2.66)	1.83(1.34-2.50)	1.78(1.31-2.41)
P_{trend}			<0.001	<0.001	
**ETS at work**					
			**Both men and women**		
No	1,060(91.5)	4,032(93.9)	1.00	1.00	1.00
Yes	99(8.5)	260(6.1)	1.45(1.14-1.84)	1.27(0.97-1.66)	1.25(0.96-1.63)

None	1,060(91.5)	4,032(93.9)	1.00	1.00	1.00
Quartile 1	22(1.9)	62(1.4)	1.35(0.83-2.21)	1.54(0.90-2.64)	1.46(0.88-2.41)
Quartile 2	14(1.2)	59(1.4)	0.90(0.50-1.62)	0.86(0.46-1.62)	0.88(0.50-1.57)
Quartile 3	24(2.1)	74(1.7)	1.23(0.78-1.97)	1.10(0.67-1.81)	1.09(0.68-1.74)
Quartile 4	39(3.4)	65(1.5)	2.28(1.53-3.41)	1.54(0.98-2.41)	1.48(0.96-2.27)
P_{trend}			<0.001	0.089	
			**Men**		
No	449(89.4)	2,258(93.0)	1.00	1.00	1.00
Yes	53(10.6)	170(7.0)	1.57(1.13-2.17)	1.41(0.98-2.02)	1.38(0.97-1.96)
None	449(89.4)	2,258(93.0)	1.00	1.00	1.00
Quartile 1	9(1.8)	39(1.6)	1.16(0.56-2.41)	1.40(0.64-3.08)	1.29(0.65-2.56)
Quartile 2	8(1.6)	45(1.9)	0.89(0.42-1.91)	0.74(0.33-1.66)	0.80(0.40-1.60)
Quartile 3	13(2.6)	43(1.8)	1.52(0.81-2.85)	1.44(0.72-2.85)	1.34(0.72-2.47)
Quartile 4	23(4.6)	43(1.8)	2.69(1.61-4.51)	2.07(1.16-3.69)	1.86(1.09-3.17)
P_{trend}			<0.001	0.021	
			**Women**		
No	611(93.0)	1,774(95.2)	1.00	1.00	1.00
Yes	46(7.0)	90(4.8)	1.48(1.03-2.14)	1.13(0.75-1.70)	1.12(0.76-1.66)
None	611(93.0)	1,774(95.2)	1.00	1.00	1.00
Quartile 1	12(1.8)	13(0.7)	2.68(1.22-5.90)	2.34(0.99-5.56)	1.84(0.88-3.84)
Quartile 2	13(2.0)	32(1.7)	1.18(0.62-2.26)	0.99(0.49-1.98)	0.99(0.53-1.84)
Quartile 3	6(0.9)	21(1.1)	0.83(0.33-2.07)	0.84(0.32-2.17)	0.89(0.40-1.95)
Quartile 4	15(2.3)	24(1.3)	1.82(0.95-3.48)	0.98(0.48-2.00)	0.98(0.52-1.85)
P_{trend}			0.086	0.992	

[a] i.e., non-active smokers. [b] Adjusted for sex (male = 1, female = 0, except when stratified by sex), age (continuous), education level (illiteracy = 1, primary = 2, middle = 3, high or college = 4), income 10 years ago (Yuan/year, continuous), body mass index (continuous), family history of esophagus cancer (yes = 1, no = 0), county of residence (Dafeng = 1, Ganyu = 2, Chuzhou = 3, Tongshan = 4), ethanol consumption (ml/week, continuous), passive smoking from home (yes = 1, no = 0, except for all variables in ETS at home), and passive smoking from work (yes = 1, no = 0, except for all variables in ETS at work).

Table S5. Combined effects of environmental tobacco smoke at home and at work using the lifetime exposure intensity as the exposure. The lifetime exposure intensity was calculated by multiplying the exposure degree by the exposure duration and adding the exposure from different family members or different workplaces. The lifetime exposure intensity was divided into five groups, including no exposure to ETS, and other four groups by the quartile of the exposure intensity to ETS in the control group.

Variables	Case N (%)	Control N (%)	Crude OR(95%CI)	Adjusted OR(95%CI)[a]	SB-Adjusted OR(95%CI)[a]
**Smokers and Non-Smokers[b]**					
No smoking at both home and work	1,522(51.3)	5,171(64.5)	1.00	1.00	1.00
Any exposure to smoking at home and/or work	1,447(48.7)	2,848(35.5)	1.73(1.59-1.88)	1.44(1.32-1.59)	1.44(1.31-1.58)
PAF (95% CI) [1]			15.0% (10.3%-18.9%)		
**Non-smokers[b] only**					
No smoking at both home and work	704(60.7)	3,186(74.2)	1.00	1.00	1.00
Any exposure to smoking at home and/or work	455(39.3)	1,106(25.8)	1.86(1.63-2.13)	1.57(1.35-1.83)	1.56(1.35-1.82)
PAF (95% CI) [1]			12.1% (8.8%-19.8%)		
**Smokers and Non-Smokers[b]**					
No smoking at both home and work	1,522(51.3)	5,171(64.5)	1.00	1.00	1.00
Smoking at either home or work	1,147(38.6)	2,366(29.5)	1.65(1.50-1.80)	1.38(1.25-1.52)	1.37(1.24-1.51)
Smoking at both home and work	300(10.1)	482(6.0)	2.12(1.81-2.47)	1.80(1.52-2.14)	1.79(1.51-2.12)
P_{trend}			<0.001	<0.001	
**Non-smokers[b] only**					
No smoking at both home and work	704(60.7)	3,186(74.2)	1.00	1.00	1.00
Smoking at either home or work	389(33.6)	977(22.8)	1.80(1.56-2.08)	1.51(1.29-1.77)	1.50(1.28-1.76)
Smoking at both home and work	66(5.7)	129(3.0)	2.32(1.70-3.15)	2.07(1.48-2.89)	1.99(1.44-2.75)
P_{trend}			<0.001	<0.001	
**Smokers and Non-Smokers[b]**					
None	1,522(51.3)	5,171(64.5)	1.00	1.00	1.00
Quartile 1	330(11.1)	681(8.5)	1.65(1.43-1.90)	1.44(1.23-1.68)	1.43(1.22-1.67)
Quartile 2	339(11.4)	736(9.2)	1.57(1.36-1.80)	1.30(1.12-1.51)	1.30(1.11-1.51)
Quartile 3	352(11.9)	716(8.9)	1.67(1.45-1.92)	1.43(1.23-1.67)	1.43(1.23-1.66)
Quartile 4	426(14.3)	715(8.9)	2.03(1.77-2.31)	1.62(1.40-1.87)	1.61(1.39-1.86)
P_{trend}			<0.001	<0.001	
**Non-smokers[b] only**					
None	704(60.7)	3,186(74.2)	1.00	1.00	1.00
Quartile 1	80(6.9)	268(6.2)	1.35(1.04-1.76)	1.42(1.07-1.88)	1.40(1.06-1.84)
Quartile 2	106(9.1)	272(6.3)	1.76(1.39-2.24)	1.51(1.17-1.96)	1.49(1.15-1.92)
Quartile 3	124(10.7)	287(6.7)	1.96(1.56-2.45)	1.55(1.21-1.98)	1.52(1.20-1.94)
Quartile 4	145(12.5)	279(6.5)	2.35(1.89-2.92)	1.79(1.41-2.28)	1.76(1.39-2.23)
P_{trend}			<0.001	<0.001	

[a] Adjusted for sex (male = 1, female = 0, except when stratified by sex), age (continuous), education level (illiteracy = 1, primary = 2, middle = 3, high or college = 4), income 10 years ago (Yuan/year, continuous), body mass index (continuous), family history of esophagus cancer (yes = 1, no = 0), county of residence (Dafeng = 1, Ganyu = 2, Chuzhou = 3, Tongshan = 4), pack-year of smoking (continuous, except for only non-smokers), and ethanol consumption (ml/week, continuous).[b] i.e., non-active smokers.
